# Ingestion of amoxicillin–clavulanic acid at therapeutic concentration during blood meal impacts *Aedes aegypti* microbiota and dengue virus transmission

**DOI:** 10.1038/s41598-024-64221-2

**Published:** 2024-06-13

**Authors:** Margot Garcia--Van Smévoorde, Elodie Calvez, Isaure Quétel, Christelle Dollin, Sébastien Breurec, Anubis Vega-Rúa

**Affiliations:** 1 Vector Control Research Laboratory, Transmission Reservoir and Pathogens Diversity Unit, Institut Pasteur de La Guadeloupe, 97139 Les Abymes, Guadeloupe France; 2 Microbial Ecosystems Interaction Laboratory, Transmission Reservoir and Pathogens Diversity Unit, Institut Pasteur de La Guadeloupe, 97139 Les Abymes, Guadeloupe France; 3 Department of Clinical Microbiology, University Hospitals of Guadeloupe, 97159 Pointe-À-Pitre/Les Abymes, Guadeloupe France; 4 Faculty of Medecine Hyacinthe Bastaraud, University of the Antilles, 97110 Pointe-À-Pitre, Guadeloupe France; 5 INSERM 1424, Center for Clinical Investigation, University Hospital Center of Guadeloupe, 97139 Les Abymes, Guadeloupe France; 6 Pathogenesis and Control of Chronic and Emerging Infections, INSERM, Etablissement Français du Sang, University of Montpellier, 34394 Montpellier, France

**Keywords:** Entomology, Viral transmission

## Abstract

Dengue virus (DENV), mainly transmitted by *Aedes aegypti* mosquitoes, is the most prevalent arbovirus worldwide, representing a public health problem in tropical and subtropical countries. In these areas, antibiotic consumption rises which may impact both mosquito microbiota and dengue transmission. Here, we assessed how the ingestion by *Ae. aegypti* of therapeutic concentrations of amoxicillin–clavulanic Acid association (Amox/Clav), a broad-spectrum antibiotic used to treat febrile symptoms worldwide, impacted its microbiota. We also evaluated whether simultaneous ingestion of antibiotic and DENV impacted *Ae. aegypti* ability to transmit this virus. We found that Amox/Clav ingestion impacted microbiota composition in *Ae. aegypti* and we confirmed such impact in field-collected mosquitoes. Furthermore, we observed that Amox/Clav ingestion enhanced DENV dissemination and transmission by this mosquito at 21 days post-DENV exposure. These findings increase our understanding of factors linked to human hosts that may influence dengue transmission dynamics in regions with mass-drug administration programs.

## Introduction

Dengue, the most prevalent viral infection in tropical and subtropical countries is caused by dengue virus (DENV)^1,2^, an arbovirus transmitted predominantly by the bite of an infected *Aedes aegypti* mosquito^3^. Since more than 50 years, dengue incidence rate has been multiplied by 30, and today more than 3.9 billion people around the world are at risk to be infected by this virus. In 2023, more than 4.5 million of cases were reported in the world, including 4,000 deaths^1^. Currently, effective antiviral agents or vaccines are not available, or are still under development^1,4^. DENV transmission by *Ae. aegypti* is shaped by multiple parameters that can be external to the mosquito such as breeding sites, nutriment availability, climatic conditions (i.e. temperature humidity), or internal factors like vector immunity, the presence of insect specific viruses, and microbiota^5^. The microbiota of *Ae. aegypti* mosquitoes is acquired via vertical transmission (from mother to offspring)^6^ and a trans-stage (horizontal) transmission from larvae to adults, in which the microbiota from breeding sites plays a crucial role^7,8^. Then, the microbiota of *Ae. aegypti* adults is greatly influenced by water, nectar, blood and virus consumption^9^. Recent studies have shown that mosquito microbiota can modulate vector competence through (i) activation of innate immune response^10^, (ii) the release of metabolites that directly impact pathogen survival as well as its ability to infect the mosquito^8,10^ and (iii) modulation of physical barriers in midgut epithelial cells^11,12^. The blood source can also influence the gut bacterial population composition in *Ae. aegypti*^13^ and possibly influence pathogen acquisition and transmission by this vector.

In malaria endemic zones, antibiotic consumption has been shown to impact the interactions between the parasite and *Anopheles* mosquitoes^14^. Interestingly, the ingestion at therapeutic concentrations of Penicillin, a narrow spectrum antibiotic, during a blood meal impacted *Anopheles gambiae* microbiota and increased its survival, fecundity and susceptibility to *Plasmodium falciparum* infections; all of which contribute to an increased transmission of the parasite^15^. In another study, the ingestion of broad-spectrum antibiotics such as amoxicillin in combination with clavulanic acid (Amox/Clav) modified *An. gambiae* microbiota composition either in presence or absence of *Plasmodium falciparum* gametocytes^16^. Amox/Clav is commonly used as empiric therapy for many of the world health organization priority infectious syndromes in adults and children, such as community acquired pneumonia or urinary tract infections^17,18^. Beyond the impact on malaria transmission previously shown, the extensive use of this antibiotic worldwide^19^ raises also concerns regarding arboviruses transmission (especially DENV) by *Aedes* mosquitoes. However, this question has been poorly investigated and how antibiotics ingestion during a blood meal impact both *Ae. aegypti* microbiota and its ability to transmit arboviruses remains unknown. For these reasons, we investigated here for the first time how the ingestion of a blood meal, supplemented with therapeutic concentrations of Amox/Clav, impacted *Ae. aegypti* gut microbiota in both field-collected and laboratory-reared mosquitoes. Then, using laboratory-reared mosquitoes, we evaluated the impact of simultaneous ingestion of Amox/Clav and DENV on both *Ae. aegypti* microbiota and vector competence for this virus.

## Results

### Ingestion of Amox/Clav did not significantly impact midgut bacterial abundance and diversity in *Ae. aegypti*

**Figure 1:**
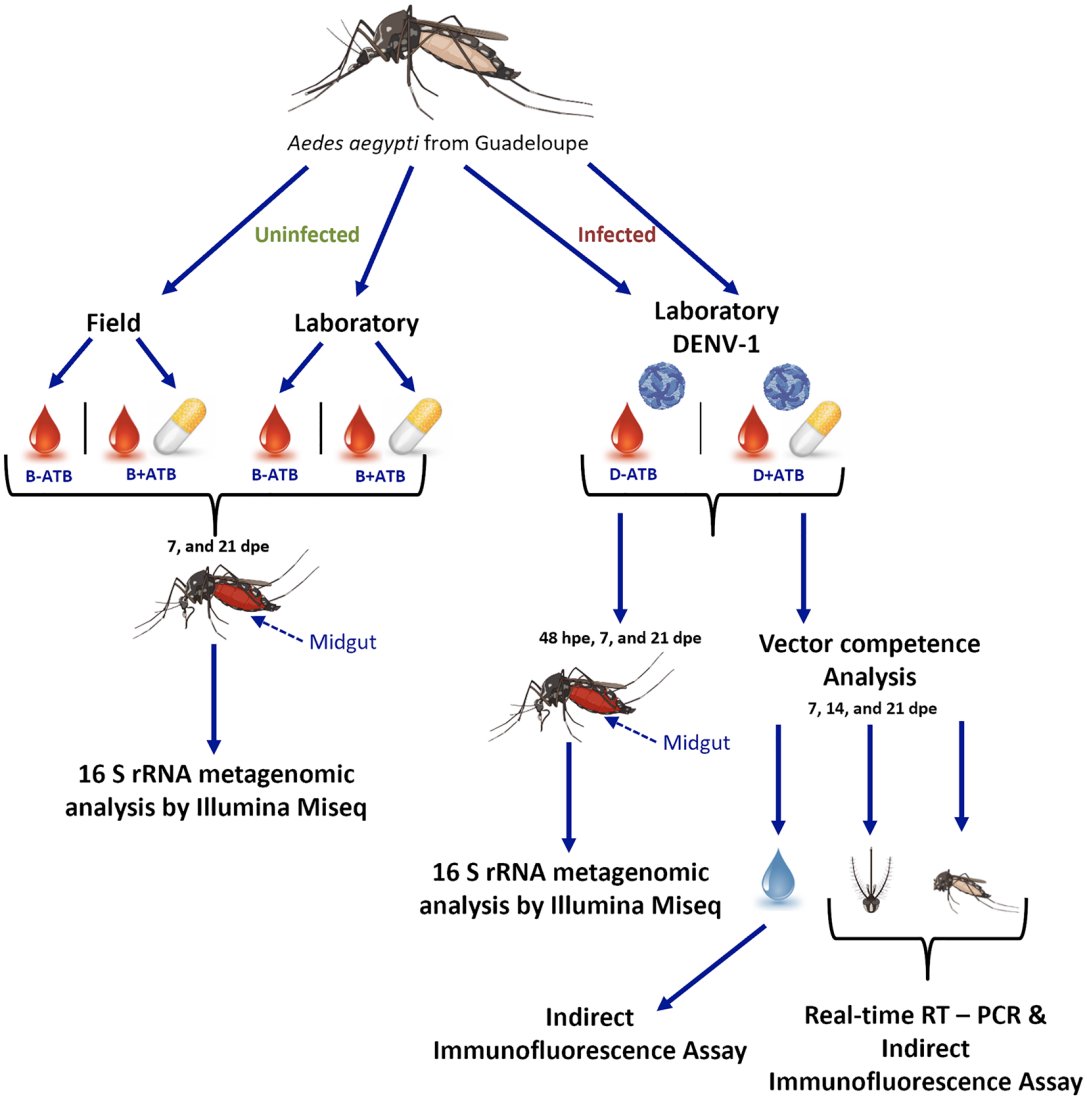
Experimental design for bacterial microbiota and vector competence analyses. One week-old *Ae. aegypti* females reared either in their breeding site water (field) or in dechlorinated tap water supplemented with rabbit food (laboratory) were blood fed either in presence or absence of Amoxicillin/ Clavulanic Acid combination (Amox/Clav). Midguts were dissected at 7 and 21 days post-exposure (dpe) before metagenomics analysis. In addition, females were infected with DENV-1 with or without the presence of the antibiotic Amox/Clav. Digestive tracts were dissected at 48 h, 7 and 21 dpe for metagenomics analysis. Individual mosquito bodies, heads and saliva were also collected at 7, 14 and 21 dpe to respectively estimate infection rate, dissemination rate, transmission rate and transmission efficiencies.

To investigate the influence of Amox/Clav ingestion on *Ae. aegypti* midgut microbiota, females that breed either in their breeding site water without diet addition (hereafter called “field condition”) or in dechlorinated tap water supplemented with rabbit food (hereafter called “laboratory condition”) were used. Blood meals were separately proposed with (B + ATB) and without the antibiotic (B−ATB), and bacterial midgut microbiota was determined at 7 and 21 days post exposure (dpe) (Fig. 1).

**Figure 2:**
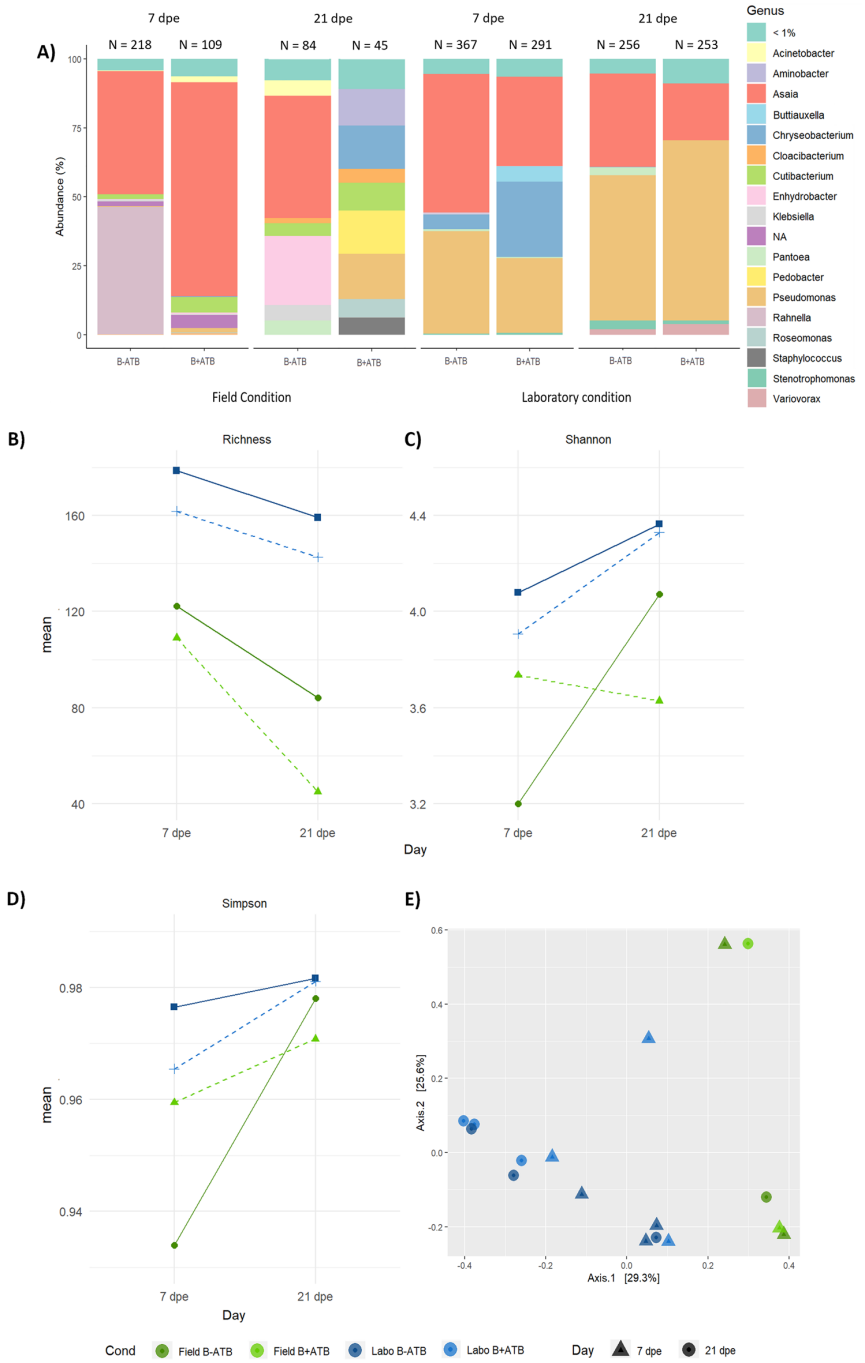
Impact of Amox/Clav ingestion during blood meal on *Ae. aegypti* bacterial microbiota. Bacterial microbiota was determined on females reared in their breeding site water (field condition) or in dechlorinated tap water supplemented with rabbit food (laboratory condition) either in presence (B + ATB) or in absence (B−ATB) of the antibiotic in the blood meal. Genera abundance (**A**), alpha diversity (Richness (**B**), Shannon (**C**), and Simpson (**D**) indices, and beta diversity (**E**) were estimated at 7 and 21 days after the blood meal. The number of amplicon simple variants (ASVs) is indicated above each barplot.

The amplicon simple variants (ASVs) abundance (1,109 ASVs) in samples decreased from 7 to 21 dpe for all conditions. In general, a higher number of ASVs was associated to samples from laboratory condition (7 dpe: 367 ASVs; 21 dpe: 256 ASVs) compared to the field condition (7 dpe: 218 ASVs, 21 dpe: 84 ASVs) (Fig. 2a). Accordingly, alpha diversity was significantly higher in laboratory condition compared to field condition (Fig. 2b, c; P = 0.0027, Shannon index; P = 0.0181; Simpson index) and at 7dpe compared to 21 dpe (Fig. 2b, c; P = 0.0264, Shannon index; P = 0.0261; Simpson index).

Whatever the day post exposure, beta diversity significantly differed between the two rearing conditions (Fig. 2e; R^2^ = 0.29, *P* = 0.003) and differences were also observed between the two days post exposure (Fig. 2e; R^2^ = 0.13, P = 0.005).

Regarding the impact of Amox/Clav ingestion, less ASVs were recorded in mosquitoes ingesting the antibiotic (B + ATB) when compared to the controls (B−ATB), this effect being more marked in mosquitoes from field condition (7 dpe: 109 ASVs for B + ATB against 218 for B−ATB; 21 dpe: 45 ASVs for B + ATB against 84 for B−ATB) (Fig. 2a). When considering all day post exposure, alpha diversity (Fig. 2b, P = 0.0431, Observed richness; Fig. 2c, P = 0.029, Shannon index) and beta diversity (Fig. 2e; R^2^ = 0.29, P = 0.003) were significantly different between mosquitoes that ingested the antibiotic and the control group. However, antibiotic ingestion did not impact alpha diversity (Fig. 2b, P = 0.509, Observed richness; Fig. 2c, P = 0.884, Shannon; Fig. 2d, P = 0.795, Simpson indices) nor beta diversity (Fig. 2e; R^2^ = 0.05, P = 0.68) at a given dpe.

### Ingestion of Amox/Clav during blood meal impacted microbiota composition in *Ae. aegypti*

Despite Amox/Clav ingestion did not greatly impact bacterial diversity at a given dpe, an impact on microbiota composition was observed. Overall, microbiota in samples was dominated by *Asaia* followed by *Pseudomonas* (Fig. 2a). *Asaia* was the most abundant and present bacteria across samples in both conditions (field and laboratory) and at the two dpe (relative abundance between 20.5 and 77.6%), while *Pseudomonas* was present in all laboratory conditions (relative abundance between 27.0 and 65.4%). In the field condition, *Pseudomonas* was only present after Amox/Clav ingestion but in minor proportion (1,6% at 7 dpe and 16,4% at 21 dpe).

Other bacteria genera such as *Chryseobacterium*, *Ranhella* and *Enhydrobacter* showed differences on relative abundance driven by antibiotic ingestion. *Chryseobacterium* was mainly present in conditions with Amox/Clav, like at 7 dpe in the laboratory condition (B + ATB: 27.3% against 5.3% in B−ATB) and at 21 dpe in the field condition (B + ATB: 15.6% relative abundance; absence in B−ATB). Within the field condition, *Rahnella* and *Enhydrobacter* were respectively found at 7 dpe (46.3% relative abundance) and 21 dpe (25% relative abundance) only in absence of the antibiotic, while they were not found after ingestion of Amox/Clav. Interestingly, a modification of the microbiota composition profile was observed at 21 dpe, where a highly diverse profile composed of eight abundant bacterial genera in similar proportions was observed (Fig. 2a).

### Simultaneous ingestion of Amox/Clav and DENV during blood meal did not impact bacterial abundance and diversity but influenced the relative abundance of specific genera

To study the influence of Amox/Clav ingestion on the midgut microbiota of *Ae. aegypti* in the presence or absence of DENV-1, females that bred in dechlorinated tap water supplemented with rabbit food (laboratory condition) were used. Blood meals were offered separately with (D + ATB) and without virus (D−ATB), and the midgut bacterial microbiota was determined at 48 h post-exposure (hpe), 7 and 21 dpe (Fig. 1).

**Figure 3:**
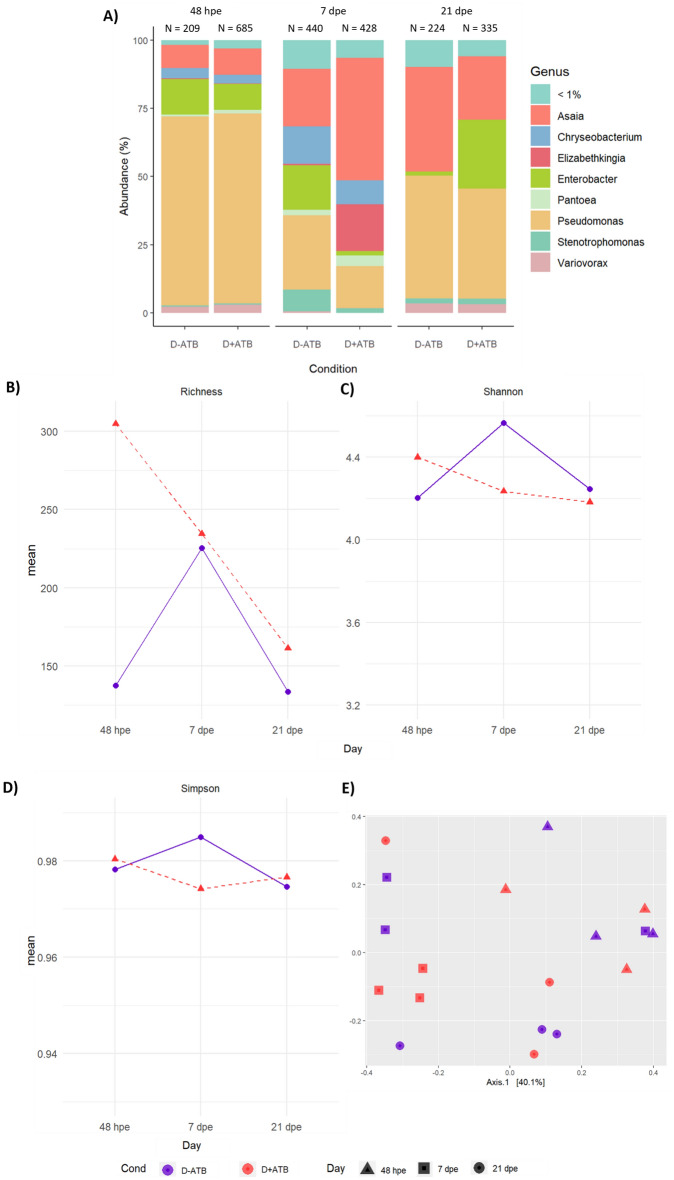
Impact of Amox/Clav ingestion during the infected blood meal on *Ae. aegypti* bacterial microbiota. The bacterial microbiota was determined for females reared in dechlorinated tap water supplemented with rabbit food (laboratory condition), either in the presence (D + ATB) or absence (D−ATB) of the antibiotic during the infection blood meal containing DENV. Genera abundance (**A**), alpha diversity (**B**) and beta diversity (**C**) were recorded at 48 h, 7 and 21 days after the blood meal. The number of single amplicon variants (ASVs) is shown above each barplot.

The ASVs abundance (1,224 ASVs) in infected samples decreased from 48 hpe to 21 dpe and differences on beta diversity were observed according to timepoints (Fig. 3e; R^2^ = 0.28, P = 0.001). A higher number of ASVs was observed in presence of the antibiotic at both 48 hpe (685 ASVs for D + ATB against 209 ASVs for D-ATB) and 21 dpe (335 ASVs for D + ATB against 224 for D−ATB condition) but no differences were observed at 7 dpe (Fig. 3a). Nevertheless, no significant difference in alpha nor beta diversity were observed according to the presence or absence of Amox/Clav in the DENV-infected blood meal (Fig. 3b–e; P > 0.05, Shannon, Observed, Simpson indices).

The most abundant genera across samples were *Pseudomonas* (between 15.4 and 69.8%) and *Asaia* (between 8.6 and 44.9%) for the two conditions tested at the three dpe (Fig. 3a). While *Pseudomonas* was predominant at 48 hpe, its relative abundance decreased at 7 dpe to later increase at 21 dpe. Conversely, *Asaia* abundance was low at 48 hpe, but then increased towards 7 and 21 dpe. In the presence of Amox/Clav, the specific presence of *Elizabethkingia* was noticed only at 7 dpe (17.2%). It is also interesting to note that while *Enterobacter* was present in both infected conditions (D + ATB and D−ATB) during the study period, its major abundance was found at 21 dpe for D + ATB condition (25.2%) (Fig. 3a).

### Ingestion of Amox/Clav during blood meal did not impact DENV infection in *Ae. aegypti*

The impact of Amox/Clav on *Ae. aegypti* vector competence was assessed on mosquito females that were artificially infected with DENV-1, either with or without antibiotics (conditions D + ATB and D−ATB, respectively). Infection, dissemination, and transmission rates were evaluated using mosquito bodies, heads and saliva, respectively. These parameters were estimated at 7, 14 and 21 dpe (Fig. 1) by viral titration. Real-time RT-PCR was also performed at the same dpe on the midgut and head samples to assess if simultaneous ingestion of Amox/Clav and DENV could impact viral replication in these mosquito compartments (Fig. 1).

**Figure 4:**
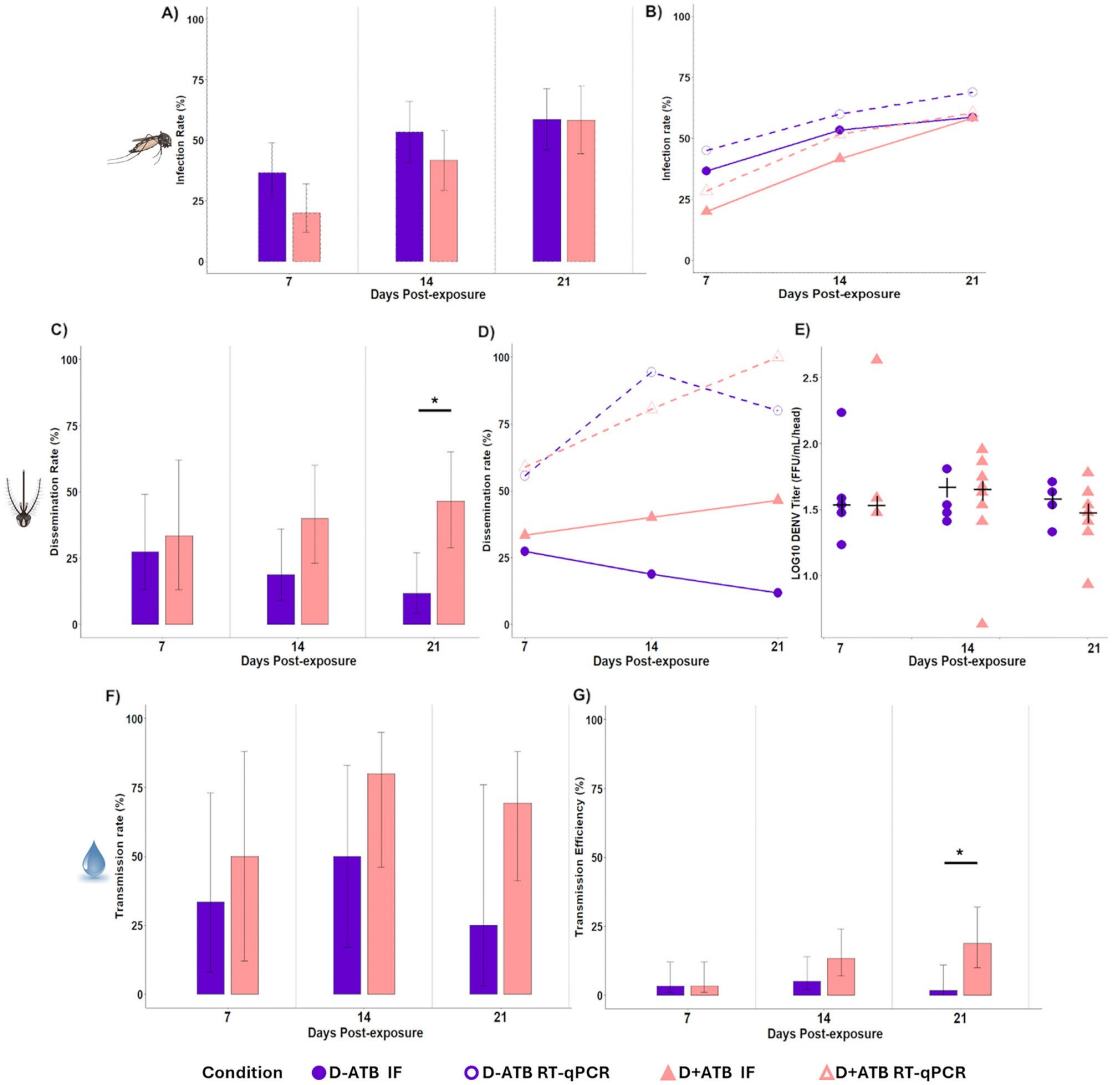
Impact of simultaneous ingestion of DENV-1 and Amox/Clavon *Ae. aegypti* vector competence at 7, 14 and 21 days after exposure. Dengue virus infection rate (**A**), dissemination rate (**C**), transmission rate (**F**), transmission efficiency (**G**) and DENV-1 viral loads (**E**). Comparison between real-time RT-PCR and viral titration estimations for infection (**B**), dissemination (**D**) in presence (D + ATB) or absence (D−ATB) of the Amox/Clav in the infectious blood meal. Infection was estimated from mosquito midguts, dissemination from heads and transmission from saliva. Error bars indicate 95% confidence intervals for each condition. Significant differences are indicated by asterisks (**P < 0.01).

Susceptibility to DENV-1 infection was observed from 7 dpe for the two conditions (D + ATB and D−ATB) and increased at 14 and 21 dpe (Fig. 4a, Supplementary data 1). At 14 dpe, infection rates (IR) were slightly higher for D−ATB condition (IR 53.3%) compared to the condition with antibiotics (D + ATB IR 41.7%). However, no significant difference was found between these two conditions whatever the dpe (P > 0.05). In addition, antibiotic ingestion does not appear to impact DENV replication within the midgut, as IR recorded at 7, 14 and 21 dpe using real-time RT-PCR data (referring to genomes) and titration (referring to infectious particles) were all similar, thus suggesting a negligible presence of defective viral particles (P > 0.05) (Fig. 4b).

### Ingestion of Amox/Clav led to higher DENV dissemination in *Ae. aegypti* at 21 dpe

Dissemination rate (DR) ranged from 27.3% (7 dpe) to 11.8% (21 dpe) for D−ATB condition and from 33.3 (7 dpe) to 46.4% (21 dpe) for D + ATB condition (Fig. 4c, Supplementary data 1). No significant differences were observed either between conditions or dpe, except at 21 dpe, where DR was significantly higher for D + ATB (46.4%) compared to D−ATB (11.8%) (P = 0.004). Similarly, real-time RT-PCR also revealed a higher number of DENV genomes in mosquito heads after ingestion of Amox/Clav at 21 dpe (P = 0.0172) (Fig. 4d).

Interestingly, we found that antibiotic ingestion enhanced DENV fitness during dissemination stage within the mosquito population since 14 dpe. In presence of the antibiotic (D + ATB), two-fold more mosquitoes had DENV genomes in their heads compared to those having infectious virus. Indeed, the proportion of mosquitoes with infected heads estimated by titration was 40% against 80.6% estimated by real-time RT-PCR at 14 dpe. Similarly, at 21 dpe, 46.4% of mosquitoes were found to disseminate DENV by titration, while 100% of them had DENV genomes in their heads according to real-time RT-PCR estimations. In absence of the antibiotic (D−ATB), this ratio increased up to six-fold, with only 18.7% of mosquitoes displaying infectious DENV in their heads despite 94.4% of them had DENV genomes at 14 dpe. A similar trend was observed at 21 dpe (D−ATB), where 11.8% had infectious DENV in heads against 80% displaying viral DENV genomes in that compartment (Fig. 4d). Beyond this effect, Amox/Clav ingestion did not impact loads of infectious particles in heads, as no significant differences were detected in presence or absence of the antibiotic whatever the dpe (P > 0.05). Viral loads ranged from 0.6–2.6 log_10_ FFU/head for D + ATB and from 1.2–2.2 log_10_ FFU/head for D−ATB (Fig. 4e).

### Ingestion of Amox/Clav during the blood meal enhanced DENV transmission by *Ae. aegypti* at 21 dpe

The analysis of mosquito saliva revealed overall higher transmission rates (TR) and efficiencies (TE) after antibiotic ingestion (D + ATB) when compared to the control (D−ATB), especially at 14 and 21 dpe (Fig. 4f, g, Supplementary data 1). Indeed, at 14 dpe, TR and TE were respectively 80.0 and 13.3% for D + ATB condition and 50 and 5% for D−ATB. Similarly, at 21 dpe, TR and TE were respectively 69.2 and 18.8% for D + ATB condition and 25.0 and 1.7% for D-ATB. No significant difference on TR were found between both conditions (P > 0,05). However, at 21 dpe, TE was significantly higher for D + ATB condition (18.7%) compared to D-ATB (1.7%) (Fisher’s test, P = 0.005).

## Discussion

In this study, we firstly investigated if the ingestion of Amox/Clav during the blood meal could affect *Ae. aegypti* microbiota by using mosquitoes that were reared either under field or laboratory conditions. Regardless the rearing condition used, our data revealed that this antibiotic did not influence gut microbiota abundance nor alpha and beta diversity in the mosquito. Gut microbiota associated to *Ae. aegypti* in our study was dominated by environmental Gram-negative bacteria belonging to *Proteobacteria*, *Bacteroidetes*, *Firmicutes* and *Actinobacteria* phyla, as previously described for mosquitoes^20^. Interestingly, most of the abundant genera identified in our samples (i.e. *Asaia, Pseudomonas, Chryseobacterium*) have been reported as naturally resistant to Amox/Clav^21–23^, which may explain the low impact of this antibiotic on the whole gut microbiota diversity except at 21 dpe, where the antibiotic was associated to a more diverse profile among abundant genera in mosquitoes from the field condition. In presence of Amox/Clav, the abundance of dominant bacteria (like *Asaia*) was disrupted, as previously observed in *Anopheles* mosquitoes^16^, which probably allowed other less competitive and opportunistic bacteria to expand. Overall, *Asaia* was the most prevalent genus across samples followed by *Pseudomonas*. *Asaia* is commonly found in field-collected or laboratory-reared mosquitoes^9,24–27^ and has shown to be abundant in mosquitoes that received a noninfectious blood meal^9^. *Pseudomonas* are also frequently found in *Aedes* mosquitoes^8,9^ and in our study, they were particularly associated to the laboratory conditions (with or without antibiotic). Their abundance has been shown to increase with sugar feeding^9^, which may explain the higher prevalence observed here at 21 dpe compared to 7 dpe. Despite the dominance of *Asaia* and *Pseudomonas,* in most samples, microbiota composition was modified after antibiotic ingestion during a non-infectious blood meal; the effect being more marked in mosquitoes from the field condition. Indeed, *Chryseobacterium* relative abundance increased in presence of the antibiotic as previously observed^15^*,* while that of *Rahnella* and *Enhydrobacter*, known to be naturally resistant to Amox/Clav^21^, decreased especially in mosquitoes from the field condition. *Chryseobacterium* (*Flavobacteriaceae*) is commonly found in mosquitoes after a blood meal^8,28,29^ and has shown to be enhanced at early dpe after ZIKV infection^9^.

When an DENV-spiked blood meal was provided, simultaneous ingestion of DENV and the antibiotic increased relative abundance of *Asaia, Elizabethkingia* and decreased *Enterobacter* prevalence at 7 dpe, while a different trend was observed at 21 dpe for *Enterobacter*. Interestingly, an anti-Plasmodium role of *Asaia* have been recognized in *Anopheles* mosquitoes^25,30^, but its possible influence on DENV transmission by *Ae. aegypti* has not been yet characterized. *E. anophelis* has been also associated with an attenuation of *Plasmodium* development in *An. gambiae*^31^, as well as with a reduction of DENV, ZIKV and CHIKV loads in vitro and that of ZIKV in vivo in *Aedes albopictus*^32^. Finally, *Enterobacter* has shown to limit the intensity of midgut infection by DENV in *Ae. aegypti*^33^.

Given the impact of Amox/Clav on bacteria genera for which an impact on pathogen transmission by mosquitoes has been described, we also investigated the influence of simultaneous ingestion of this antibiotic with DENV during the blood meal on *Ae. aegypti* vector competence for this virus. Our results highlighted a moderated infection of *Ae. aegypti* (IR < 59%) following oral DENV-1 ingestion, a limited dissemination of the virus in the vector body (DR < 46.4%) and a transmission efficiency lower than 19%. This limited DENV-1 transmission by *Ae. aegypti* was also described in oral infections at similar viral titers (i.e. 10^6^–10^7^ FFU/mL) on mosquitoes from the Caribbean^34^, Africa^35^, and South Pacific^36^.

In our experiments, antibiotic ingestion during the blood meal did not impact vector competence for DENV at early dpe. However, significant higher DR and TE were observed in presence of the antibiotic at 21 dpe. The investigation on DENV detection in mosquito heads via real-time RT-PCR and immunofluorescence also revealed that antibiotic may have enhanced DENV fitness during dissemination stage within the mosquito population. Since 14 dpe, in presence of the Amox/Clav, the proportion of mosquitoes presenting DENV genomes was two-fold higher than those containing infectious viral particles in the head, whereas in absence of the antibiotic, the proportion of mosquitoes having genomes, but no infectious virus was much more important (sixfold). Such differences may reflect a higher proportion of DENV-free defective interfering particles (DIPs) in the condition D-ATB, resulting from problems on viral replication or assembly possibly associated to mosquito immunity^37^ that should be further investigated. Previous studies demonstrated the potential role of DIPs in antiviral activity for DENV in vitro or by modelling data in mosquitoes, which could explain our observations^38,39^.

In another hand, the increase of DENV-infected mosquitoes observed during the study period, coupled with the higher dissemination observed at 21 dpe in presence of the antibiotic, could be due to an increased midgut epithelium permissiveness associated to changes on microbiota composition. Previous studies demonstrated that midgut epithelium in mosquitoes could be degraded via the secretion of specific proteins by some microbiota members as *Serratia marcescens*, thus resulting in pathogen transmission enhancement^15,40^. At 21 dpe, *Enterobacter* abundance was higher in presence of both DENV and Amox/Clav, which may suggest a potential role of this genus in promoting DENV dissemination and transmission in our setting. Interestingly, when Carlson and colleagues^33^ added *Enterobacter ludwigii* to *Ae. aegypti* immature stages, this bacterium was shown to limit midgut infection by DENV-2. Despite their methodological differences (artificial larval *Enterobacter* exposure versus natural Enterobacter presence in adult microbiota), these studies together highlight possible interactions between *Enterobacter* genus and DENV in *Ae aegypti* whose mechanisms should be further investigated. It is note worthing that *Ae. aegypti* mosquitoes have shorter average lifespan in the field (around 10–15 days)^41^ but they renew blood feeding at each gonotrophic cycle to maintain egg development. In our study, we observed already increased dissemination and ability for DENV transmission by *Ae. aegypti* during the first gonotrophic cycle, with viral loads in agreement with previous reports^42^ . Previous studies have shown that midgut permissiveness increases in case of several gonotrophic cycles during *Ae. aegypti* lifespan^43^, which coupled to antibiotic ingestion, could increase even more the risk for DENV transmission.

Taken together, our results demonstrate that the presence of Amox/Clav at a therapeutic dose during a blood meal modify *Ae. aegypti* microbiota and promotes DENV dissemination and transmission at late dpe (as observed at 21 dpe). The Amox/Clav is an antibiotic commonly used to treat febrile symptoms worldwide^19^. Since several years, antibiotic use is still rising especially in low-and middle-income countries^44,45^ from South America, Asia, Africa and South Pacific where *Ae. aegypti* mosquitoes are abundant and DENV circulation is active^2^. In this context, our study raises concerns regarding the risk of DENV emergence and spread associated with high antibiotic consumption and emphasizes the need of investigate further these human hosts-vector interactions using other models to deepen the understanding of DENV transmission dynamics.

## Material and methods

### Ethics statement

All experiments were performed in accordance with relevant guidelines and regulations and this study has been approved by internal ethics committee of the Institut Pasteur de la Guadeloupe (established since September 2015). For mosquito rearing, blood > 40 days-old for non-therapeutic use were purchased to the French Blood Agency (EFS) under a cession agreement N° PLER-01/2020/EFS-IPGUAD. For the experiments, Anubis Vega-Rúa (author of the study) provided written consent for human blood donation to artificially feed mosquitoes.

### Mosquito sampling and rearing

*Ae. aegypti* immature stages (larvae and pupae) were collected during a collection in January 2021 from ten tires (16°15′00.4ʺN 61°32′51.2ʺW) and two drums (16°15′05.3ʺN 61°32′52.0ʺW) at Lauricisque, Pointe-à-Pitre, Guadeloupe. A first batch of larvae (hereafter called “laboratory mosquitoes”) was reared in dechlorinated tap water and fed with rabbit food pellets (GMA, Baie-Mahaut, Guadeloupe). After emergence, the adults were kept in cages under controlled conditions with temperature maintained at 28 °C, 70% humidity, 12 h light/dark cycle and fed with 10% sucrose solution ad libitum*.* Twice a week, females were fed with a blood meal (see ethics statement section for more details) with an artificial feeding system (Hemotek Ltd, UK) using pig intestine membrane to obtain the first *Ae. aegypti* generation (F1) used for the experiments. A second batch or larvae referred as “field mosquitoes” was collected along with the breeding sites water that was used for their rearing. Emerging mosquitoes (F0 generation) were directly used for the experiments. The maintenance of adults after emergence was conducted as explained above for laboratory mosquitoes.

### Virus strain

The DENV-1 strain used was isolated from a patient serum in Guadeloupe in 2016 (GenBank accession number: OR486055). As described by Gutierrez-Bugallo et al.^34^, viral stocks were prepared after two passages in *Ae.* *albopictus* C6/36 cells at an MOI of 0.1 in Leibovitz 15 medium (L15; Invitrogen, USA) supplemented with 2% fetal bovine serum (FBS; Gibco, Fisher scientific, USA). The supernatants were collected after five days of incubation at 28 °C and the viral stock was kept at −80 °C. Viral titer was estimated by serial tenfold dilutions in C6/36 cells and expressed as focus-forming units (FFU)/mL.

### Experimental mosquito infection

Female mosquitoes (seven to ten days-old and not previously blood-fed) were starved 24 h before oral infection. Each capsule of the feeding system (Hemotek Ltd, UK) contained a mixture of 1.4 mL of washed rabbit erythrocytes, 700 μL of DENV-1 viral suspension (diluted in Leibovitz L15 media) and the phagostimulant adenosine triphosphate (Sigma-Aldrich, Germany) at a final concentration of 5 mM. The final virus titer of the blood meal was 10^7^ FFU/mL for all conditions. For the condition with Amox/Clav (D + ATB), the antibiotic was added at a final Amox/Clav concentration of 8.3 µg/L^18^ in the infected blood meal. Amox/Clav concentration in the blood meal was confirmed by the clinical microbiology and anti-infectives assays department at the Paris Saint-Joseph Hospital. In the control group (D−ATB), the antibiotic was replaced by the same volume of Dulbecco’s Phosphate Buffered Saline. After a twenty minutes infectious blood meal, fully engorged females were selected and placed into boxes and maintained in climatic chambers (Memmert, Schwabach, Germany) under controlled conditions (28 °C; 70% humidity; 12 h light/dark cycle) and fed ad libitum with a 10% sucrose solution.

### Microbiota analysis

To investigate the impact of Amox/Clav ingestion on *Ae. aegypti* microbiota, females were reared either in dechlorinated tap water supplemented with rabbit food pellets or in water collected from their breeding sites (see Mosquito sampling and rearing section for more details). Then, two mosquito batches of 5–7 days old mosquitoes were separately blood fed. One group received blood supplemented with the antibiotic (B + ATB condition), while the control group just received blood (B-ATB condition) (Fig. 1). At 7 dpe and 21 dpe, the digestive tracts were dissected and surface sterilized (5 min in 75% ethanol, followed by 3 washes in PBS) as previously described^15^. Pools of 10 midguts (3 replicates per condition) were stored in 300µL of Phosphate Buffered Saline (PBS) at −80 °C for further use.

To investigate the influence of simultaneous ingestion of Amox/Clav and DENV on mosquito microbiota, mosquitoes reared in laboratory tap water where orally exposed to a blood meal containing DENV-1 and Amox/Clav (D + ATB condition), while the control group received blood supplemented only with DENV-1 (D-ATB condition). DENV titer in both blood meals was set at 10^7^ FFU/mL. At 48 hpe, 7 dpe and 21 dpe digestive tracts (3 pools of 10 midguts per condition) were surface sterilized and dissected for microbiota analysis as described above.

### Bacterial DNA extraction

Before DNA extraction, 100 µL of PBS were added to the samples. Midguts were crushed with 0.5 mm wide glass beads using a bead beater (MM 400, Retsch, France) at 30 Hz for 20 s (three times) and samples were then centrifuged at 12000 rpm during 3 min. Extraction was performed using RNeasy® PowerMicrobiome® kit, following the manufacturer’s instructions (Qiagen, Hilde, Germany). The presence of 16S rDNA was confirmed using the universal primers 27F (5′ AGAGTTTGATCCTGGCTCAG 3′) and 1492R (5′ GGTTACCTTGTTACGACTT 3′) as previously described^46^ and the DreamTaq Green PCR Master mix kit (Thermoscientific, Vilnius, Lithuania). DNA from amplicons was visualized under ultraviolet light on a 1.5% electrophoresis agarose gel stained with gel red (Biotium, USA).

### Illumina MiSeq sequencing, sequence processing and analysis

Amplification of V3–V4 region of the 16S rRNA gene was conducted using the universal prokaryote-specific primers 341F (CCTACGGGNGGCWGCAG) and 785R (GACTACHVGGGTATCTAATCC)^47^. Illumina MiSeq 250-bp paired-end sequencing was performed on MiSeq v3 600-cycle cartridges with the addition of 10% PhiX for diversity, by the Biomics platform of the Institut Pasteur (Paris, France). The raw sequences obtained (fastq) were cleaned using the R package dada2^48^ implemented on a galaxy platform^49,50^. This package includes filtering, merging, clustering, chimera and singleton deletion and taxonomic ASVs assignment. Reads with an average quality lower than 2 were suppressed and the taxonomic affiliation was removed if bootstrap value was lower than 50%. Then, an abundance table was obtained containing the high-quality sequences corresponding to each ASV, their taxonomy, bootstrap values of each taxonomic assignment according to the database SILVA^51^, and finally, the number of sequences per sample corresponding to each ASVs. A total of 1,060,834 high-quality sequences (62,402 reads per sample) was retained after standardization from field and laboratory samples (n = 17), representing 1,109 ASVs. For D + ATB and D−ATB samples (n = 18), 49,187 high-quality reads were retained that were clustered into 1,224 ASVs. Overall, the sequencing ensured an adequate coverage of bacterial communities (Supplementary data 2).

### Vector competence assessment

Batches of 48–60 mosquito females were separately analyzed per condition (with and without antibiotic) at 7, 14, and 21 dpe. Each female was cold-anesthetized, and its legs and wings were removed before inserting the proboscis into a 20 µl filter tip containing 5 μl of FBS. After 20 min, the saliva collected was expelled from the tip into 45 µL of Leibovitz L15 medium and stored at -80 °C before analysis. Then, for each mosquito, head and body were individually grounded in 300 µL of Leibovitz L15 medium supplemented with 2% FBS. Subsequently, the samples were centrifuged at 10,000 rpm for 5 min at 4 °C. The supernatants were stored at −80 °C before analysis.

Vector competence was estimated via determination of the infection rate (IR), dissemination rate (DR), transmission rate (TR) and the transmission efficiency (TE). IR and TE respectively correspond to the proportion of mosquitoes whose body (abdomen and thorax) and saliva is infected among the mosquitoes tested. DR corresponds to the proportion of mosquitoes with infected head among those with infected bodies. TR corresponds to the proportion of mosquitoes with infected saliva among those with infected heads.

### Determination of sample infection status

Mosquito bodies and heads were firstly screened by real-time RT-PCR. Viral genomic RNA was extracted from 100 µl of sample homogenate using the NucleoSpin® 96 Virus kit (Macherey Nagel, Duren, Germany), following the manufacturer’s instructions. Real-time RT-PCR was conducted on a 7500 Real-Time PCR (Applied Biosystems) using primers previously described^52^ and the Superscript® III Platinum® One-step Quantitative RT-PCR System with ROX kit (Invitrogen, Carlsbad CA, USA). The thermal profile used was the following: 30 min of reverse transcription at 50 °C, 10 min of denaturation at 95 °C, 45 cycles of amplification at 95 °C for 15 s followed by 58 °C for 30 secondes. All samples with a Cq value upper than 41 were considered negative. Then, the presence of infectious viral particles was confirmed by examining focus forming units in mosquito bodies, heads and saliva. Briefly, bodies, head and saliva samples from the same individuals were serially diluted and inoculated on C6/36 cells in 96-well plates. After incubation at 28 °C for 5 days, samples were fixed with 3.6% formaldehyde, then submitted to immunofluorescence assays where they were stained with mouse anti-dengue complex monoclonal (primary antibody), clone D3-2H2-9-21 (Merck KGaA, Germany) and Alexa Fluor 488 goat anti-mouse IgG (second antibody; Life Technologies, Carlsbad, USA).

### Statistical analyses

Statistical analyses for vector competence data were conducted with R (4.1.2) (R Core Team, Vienna, Austria)^53^ to compare IR, DR, TR and TE using Fisher’s exact tests. Kruskal–Wallis analysis of variance and multiple comparison tests were used to compare DENV titers. Graphics were generated using the R packages ggplot2 and plyr^54,55^. Statistical significance for all analyses was detected if P ≤ 0.05.

Microbiota analyses were performed using R packages ggplot2^55^, phyloseq^56^, and vegan^57^. Alpha diversity was quantified with the indices of Richness, Shannon, and Simpson according to the phyloseq library and an ANOVA statistical test^58^. Beta diversity was represented with a PCoA (principal coordinate analysis) based on the Bray Curtis dissimilarity index according to the phyloseq library and a PERMANOVA^59^.

### Supplementary Information

Supplementary Information.

## Data Availability

To obtain raw data, please contact the corresponding author on reasonable request.
