# Supplementary material for: Ingestion of amoxicillin–clavulanic acid at therapeutic concentration during blood meal impacts Aedes aegypti microbiota and dengue virus transmission
Source: Sci Rep. 2024 Jun 13;14:13701. doi: 10.1038/s41598-024-64221-2 (PMC11176339; doi:10.1038/s41598-024-64221-2)
Supplement: Supplementary file 1 — Supplementary Information. [file 41598_2024_64221_MOESM1_ESM.docx]

**Supplementary data 1. Dengue 1 infection, dissemination, transmission rates and transmission efficiency in *Aedes aegypti* at 7, 14 and 21 days post-exposure (dpe) according to the presence (D+ATB) or absence (D-ATB) of Amoxicillin-Clavulanic acid association (Amox/Clav) in the blood meal.**

| **Condition** | **DPE** | **Tested** | **Infection rate** | | **Dissemination rate** | | **Transmission rate** | | **Transmission efficiency** | |
| --- | --- | --- | --- | --- | --- | --- | --- | --- | --- | --- |
|  |  |  | **N** | **%** | **N** | **%** | **N** | **%** | **N** | **%** |
| **D-ATB** | 7 | 60 | 22 | 36.7 | 6 | 27.3 | 2 | 33.3 | 2 | 3.3 |
|  | 14 | 60 | 32 | 53.3 | 6 | 18.8 | 3 | 50 | 3 | 5 |
|  | 21 | 58 | 34 | 58.6 | 4 | 11.8 | 1 | 25 | 1 | 1.7 |
| **D+ATB** | 7 | 60 | 12 | 20 | 4 | 33.3 | 2 | 50 | 2 | 3.3 |
|  | 14 | 60 | 25 | 41.6 | 10 | 40 | 8 | 80 | 8 | 13.3 |
|  | 21 | 48 | 28 | 58.6 | 13 | 46.4 | 9 | 69.2 | 9 | 18.8 |

N = number of positive samples

% = percentage of positive samples

**
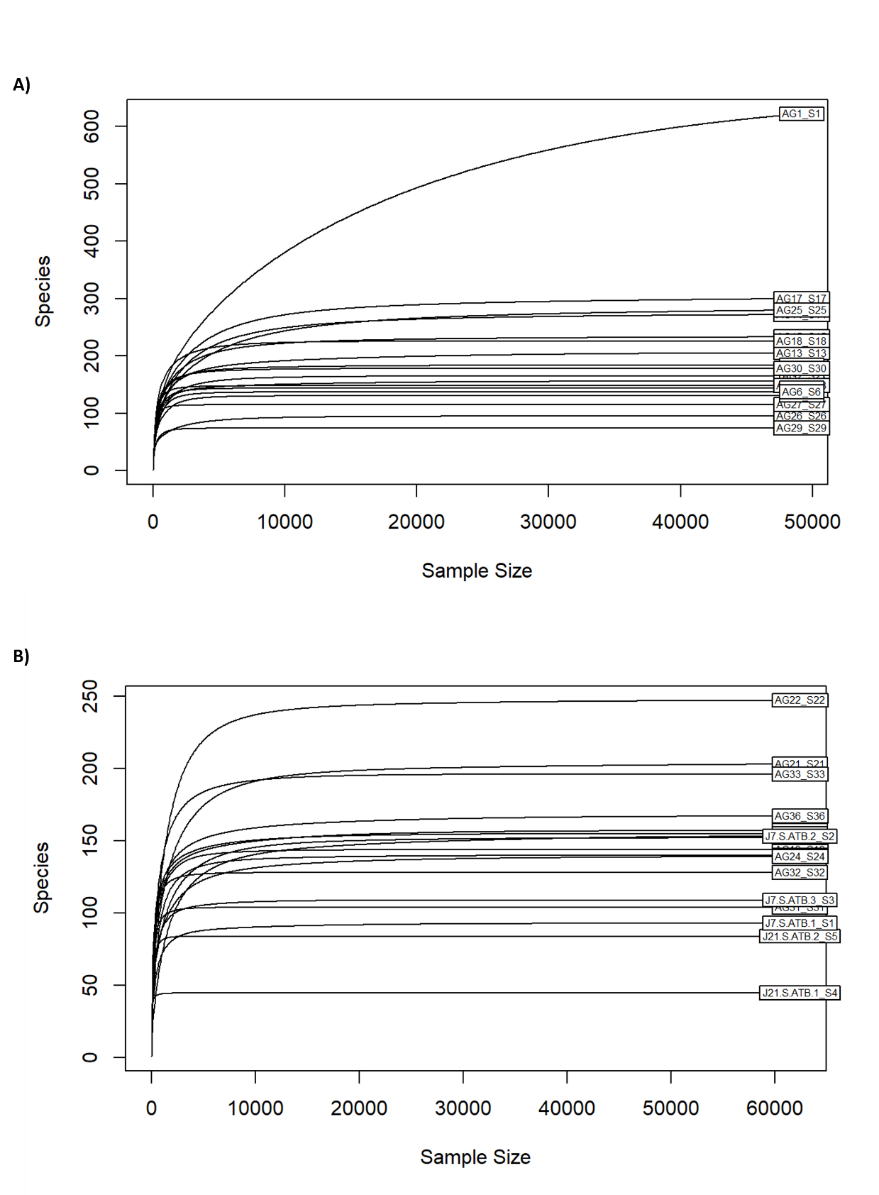
Supplementary data 2. Rarefaction curves obtained from *Aedes aegypti* midguts. Mosquitoes receiving Amoxicillin – Clavulanic Acid association (Amox/Clav) during blood meal (A), mosquitoes receiving simultaneously Amox/Clav and dengue-1 virus during the blood meal (B).**
